# Barriers and facilitators to infection prevention and control in Dutch psychiatric institutions: a theory-informed qualitative study

**DOI:** 10.1186/s12879-022-07236-2

**Published:** 2022-03-11

**Authors:** Famke Houben, Mitch van Hensbergen, Casper D. J. den Heijer, Nicole H. T. M. Dukers-Muijrers, Christian J. P. A. Hoebe

**Affiliations:** 1 Department of Sexual Health, Infectious Diseases and Environmental Health, South Limburg Public Health Service, P.O. Box 33, 6400 AA Heerlen, The Netherlands; 2 Department of Social Medicine, Care and Public Health Research Institute (CAPHRI), Faculty of Health, Medicine and Life Sciences, Maastricht University, P.O. Box 616, 6200 MD Maastricht, The Netherlands; 3 Department of Medical Microbiology, Care and Public Health Research Institute (CAPHRI), Faculty of Health, Medicine and Life Sciences, Maastricht University Medical Centre (MUMC+), P.O. Box 5800, 6202 AZ Maastricht, The Netherlands; 4 Department of Health Promotion, Care and Public Health Research Institute (CAPHRI), Faculty of Health, Medicine and Life Sciences, Maastricht University, P.O. Box 616, 6200 MD Maastricht, The Netherlands

**Keywords:** Infection prevention and control, Antimicrobial resistance, Healthcare-associated infection, Nosocomial infection, Mental health care, Psychiatry, Qualitative study

## Abstract

**Background:**

The unique characteristics of psychiatric institutions contribute to the onset and spread of infectious agents. Infection prevention and control (IPC) is essential to minimise transmission and manage outbreaks effectively. Despite abundant studies regarding IPC conducted in hospitals, to date only a few studies focused on mental health care settings. However, the general low compliance to IPC in psychiatric institutions is recognised as a serious concern. Therefore, this study aimed to assess perceived barriers and facilitators to IPC among professionals working at psychiatric institutions, and to identify recommendations reported by professionals to improve IPC.

**Methods:**

A descriptive, qualitative study involving 16 semi-structured interviews was conducted (before COVID-19) among professionals from five Dutch psychiatric institutions. The interview guide and data analysis were informed by implementation science theories, and explored guideline, individual, interpersonal, organisational, and broader environment barriers and facilitators to IPC. Data was subjected to thematic analysis, using inductive and deductive approaches. This study followed the Consolidated criteria for Reporting Qualitative research (COREQ) guidelines.

**Results:**

Our findings generated six main themes: (1) patients’ non-compliance (strongly related to mental illness); (2) professionals’ negative cognitions and attitude towards IPC and IPC knowledge deficits; (3) monitoring of IPC performance and mutual professional feedback; (4) social support from professional to patient; (5) organisational support and priority; and (6) financial and material resource limitations (related to financial arrangements regarding mental health services). The main recommendations reported by professionals included: (1) to increase awareness towards IPC among all staff members, by education and training, and the communication of formal agreements as institutional IPC protocols; (2) to make room for and facilitate IPC at the organisational level, by providing adequate IPC equipment and appointing a professional responsible for IPC.

**Conclusions:**

IPC implementation in psychiatric institutions is strongly influenced by factors on the patient, professional and organisational level. Professional interaction and professional-patient interaction appeared to be additional important aspects. Therefore, a multidimensional approach should be adopted to improve IPC. To coordinate this approach, psychiatric institutions should appoint a professional responsible for IPC. Moreover, a balance between mental health care and IPC needs is required to sustain IPC.

**Supplementary Information:**

The online version contains supplementary material available at 10.1186/s12879-022-07236-2.

## Background

Psychiatric institutions play a crucial role in the support and treatment of patients with mental illness while ensuring a psychologically and physically safe environment. However, these institutions are vulnerable to the emergence and spread of infections, including healthcare-associated infections (HAIs) and infectious diseases such as coronavirus disease 2019 (COVID-19) [1–5]. Several studies have reported high prevalence rates for infectious diseases in psychiatric inpatient populations, ranging between 21 and 38% for hepatitis B and C and 10% for human immunodeficiency virus (HIV) [6, 7]. Nevertheless, data on the prevalence of HAIs, including multidrug-resistant microorganisms, in psychiatric facilities are scarce [8]. Recently, the issue of nosocomial infections in psychiatric institutions has received an increasing amount of attention due to large-scale COVID-19 outbreaks within these facilities [9–11].

Inpatient or residential psychiatric settings present unique challenges for the emergence and spread of contagious pathogens due to communal living environments, the patient population, frequent patient-turnover and close staff-patient contact [3, 5, 6, 9, 12].

Infection prevention and control (IPC) reduces the transmission risk of (resistant) microorganisms, thereby preventing patients and healthcare workers (HCWs) from avoidable infections and antimicrobial resistance (AMR) [13, 14]. IPC includes various measures such as hand hygiene, isolation precautions, the use of personal protective equipment as gloves and masks, and disinfection of equipment and the environment.

The implementation of IPC in psychiatric settings, however, may be difficult due to patient-related factors such as lack of cooperation and motivation, which often result from cognitive limitations or severe mental illness [3]. Likewise, implementing IPC can be difficult as necessary equipment, such as alcohol-based hand sanitisers, are often not available because of patient safety reasons [5]. Moreover, factors on the care sector level may also hinder IPC. Mental health care facilities promote social interaction, group activity and freedom of movement within the institutional environment, which pose significant dilemmas in terms of IPC [3, 15]. Additionally, the general low adherence to IPC measures of mental health professionals is a serious concern [16, 17]. This indicates the need to determine the factors that impede or facilitate IPC implementation in psychiatric settings.

Although there is abundant literature on IPC in hospital settings, there are few studies addressing IPC implementation in psychiatric institutions [16, 17]. Current studies mainly focus on patient and care sector-related factors influencing IPC in psychiatric settings. However, factors which impact adherence to, and the organisation of IPC may also occur on other levels, such as the guideline, professional, interpersonal, organisational, and societal level [18]. This illustrates the need for a comprehensive assessment of factors on multiple levels that facilitate or impede IPC implementation in psychiatric settings.

The identification of barriers and facilitators to implementation is most effective when embedded in theory [19]. By conducting an in-depth theoretical analysis of the factors impeding or facilitating implementation, the probability of developing a successful intervention will increase. Moreover, an intervention is more likely to be successful when users are involved during the development [19].

This theory-informed study aims to assess perceived barriers and facilitators to IPC among professionals working at psychiatric institutions, and to identify recommendations reported by professionals to improve IPC. With the results of this study, psychiatric institutions will be enabled to optimise the implementation of IPC in their own setting.

## Methods

### Design

We used a descriptive qualitative design [20, 21]. Semi-structured interviews were conducted to determine perceived factors facilitating or impeding IPC. Implementation science theories informed the study design. The COnsolidated criteria for REporting Qualitative research (COREQ) guidelines [22] were followed for data reporting [See Additional file 1].

### Theory

Successful implementation of new practices, such as IPC, depend on behavioural and organisational change [23]. Many theories and models have been developed to provide a better understanding of how and why implementation succeeds or fails. Examples of these theories are behaviour change theories [24–26], socio-ecological models [27], theories that identify levels on which barriers and incentives to change in healthcare occur [23, 28, 29], and theories that incorporate characteristics of the innovation (in our case guidelines) [29, 30].

Our pre-proposed theoretical framework for factors influencing IPC [18] synthesised these relevant implementation science theories and adapted them to qualitative findings. The framework delineates levels on which factors influencing IPC occur. The framework includes the following levels: guideline, individual, interpersonal, organisational, community (in our case care sector), and societal level. The individual level comprises the patient and professional level. The interpersonal level includes professional interaction, professional-patient interaction, and patient interaction. Each level encompasses various factors, such as procedural clarity and compatibility on the guideline level, attitude and knowledge on the individual level, social norm and social support on the interpersonal level, and materials/equipment on the organisational level. The framework provides a basis for analysing which factors and mechanisms are working for (facilitators) or against (barriers) IPC implementation.

### Participant selection

Participants were 16 professionals from five psychiatric institutions in the Netherlands. As we aimed to obtain exhaustive insights into different perceptions and needs in the mental health care setting, we intended to include professionals from various layers of the organisation. Participants were selected by snowball sampling [31, 32]. Initial recruitment started by contacting a contact person in five psychiatric institutions in the southern part of the Netherlands (Limburg and Brabant). This contact person recruited participants within their respective organisation. After receiving contact details of eligible participants from contact persons, study invitations were extended via e-mail, telephone or during a face-to-face meeting, supplemented by an elaboration on the purpose and content of the study. Interview participants were asked to recruit future participants among their co-workers. When professionals did not respond to earlier invitations, up to two reminders were sent. The selection of participants continued until data saturation was attained [33]. Previous studies have recommended a minimum sample size of at least 12 to reach data saturation [34, 35], for which we aimed in this study.

### Data collection

A total of 16, individual one-session semi-structured interviews were conducted between February and December 2019 (before COVID-19) with participants at their work location. Written informed consent was obtained prior to the interviews. MvH (PhD student), MK (infection control professional) and MD (junior researcher) conducted the interviews. All interviewers were trained and experienced in conducting interviews and qualitative research. No relationship was established between the interviewers and participants prior the study. Before the start of the interviews, the interviewers introduced themselves as researchers, including the infection control professional, to limit receiving socially desirable answers. A topic guide consisting of 23 questions was used to guide the interviews [See Additional file 2], developed by CdH (PhD and MD, physician specialised in infection disease control) and MvH. The topic guide questions were informed by major constructs included in implementation science theories [23–30]. The guide was reviewed by, and pilot tested among four healthcare professionals, including key informants regarding IPC and a mental health professional (who was not a participant of the study), prior to data collection. To ensure applicability even more, at the end of the first four interviews, participants were asked about any themes or topics they missed during the interview. Nonetheless, no further changes were made to the data collection instrument. The main topics included descriptive data of the professionals such as occupation, age (reported as 5-year categories to ensure confidentiality), years of experience; their attitude and perceptions towards IPC; the role of IPC in their daily work; social influences regarding IPC; the role of IPC at the organisational level; and recommendations to improve IPC. All interviews were audio-recorded and transcribed verbatim by a professional transcription service.

### Data analysis

Transcripts were coded using ATLAS.ti 8.4.2 software for qualitative analysis. Data were coded both inductively and deductively, using thematic analysis [36, 37]. We employed a realist approach, meaning we focused on the manifest rather than latent interview content [38]. The coding process followed the method of Braun and Clarke [36]. First, each entire transcript was read to obtain an overall understanding of the text and to familiarise with participants’ perceptions and experiences regarding IPC. Secondly, initial codes representing a specific barrier or facilitator were identified throughout all transcripts. The initial coding frame was guided by our pre-proposed integrated theoretical framework [18] and its underlying implementation science theories [23–30]. Afterwards, initial codes were synthesised into wider themes which revealed patterns in the data resulting in a thematic map of the analysis, bringing together the extracted themes and codes therein. The barriers and facilitators within each theme were subsequently categorised into their levels of influence: guideline, patient, professional, professional interaction, professional-patient interaction, patient interaction, organisational, community, and societal level [18]. The coding process was iterative and conducted until no new codes appeared. An example of the coding process is provided in Additional file 3. The coding process was independently performed by two researchers (FH, PhD student; MD), complemented by peer review of a third researcher (MvH). Discrepancies were discussed in the expert-group until consensus was reached.

## Results

Out of 22 professionals from five psychiatric institutions invited, 16 (73%) participated in the study. Reasons for non-participation were time constraints in all. The final sample consisted of ten women and six men, including ten professionals who work with patients (social worker, nursing assistant, nurse, physician, psychiatrist) and six managerial professionals (quality assurance officer, supervisor, manager). On average, participants were 47 years old (range 25–66 years). The interviews lasted 41 min on average (range 20–62 min). Data saturation [33] was confirmed to be reached after data analysis, since no new information regarding barriers, facilitators or recommendations emerged after the fourteenth interview.

### Categories and themes

Qualitative analysis generated six main themes: (1) Patients’ non-compliance (strongly related to mental illness); (2) professionals’ negative cognitions and attitude towards IPC and IPC knowledge deficits; (3) monitoring of IPC performance and mutual professional feedback; (4) social support from professional to patient; (5) organisational support and priority; and (6) financial and material resource limitations (related to financial arrangements regarding mental health services). These main themes covered barriers and/or facilitators within their respective levels, and were identified based on the overarching interpretation of the data and associations across levels.

### Integrated theoretical framework for factors influencing IPC in mental health care settings

**Fig. 1:**
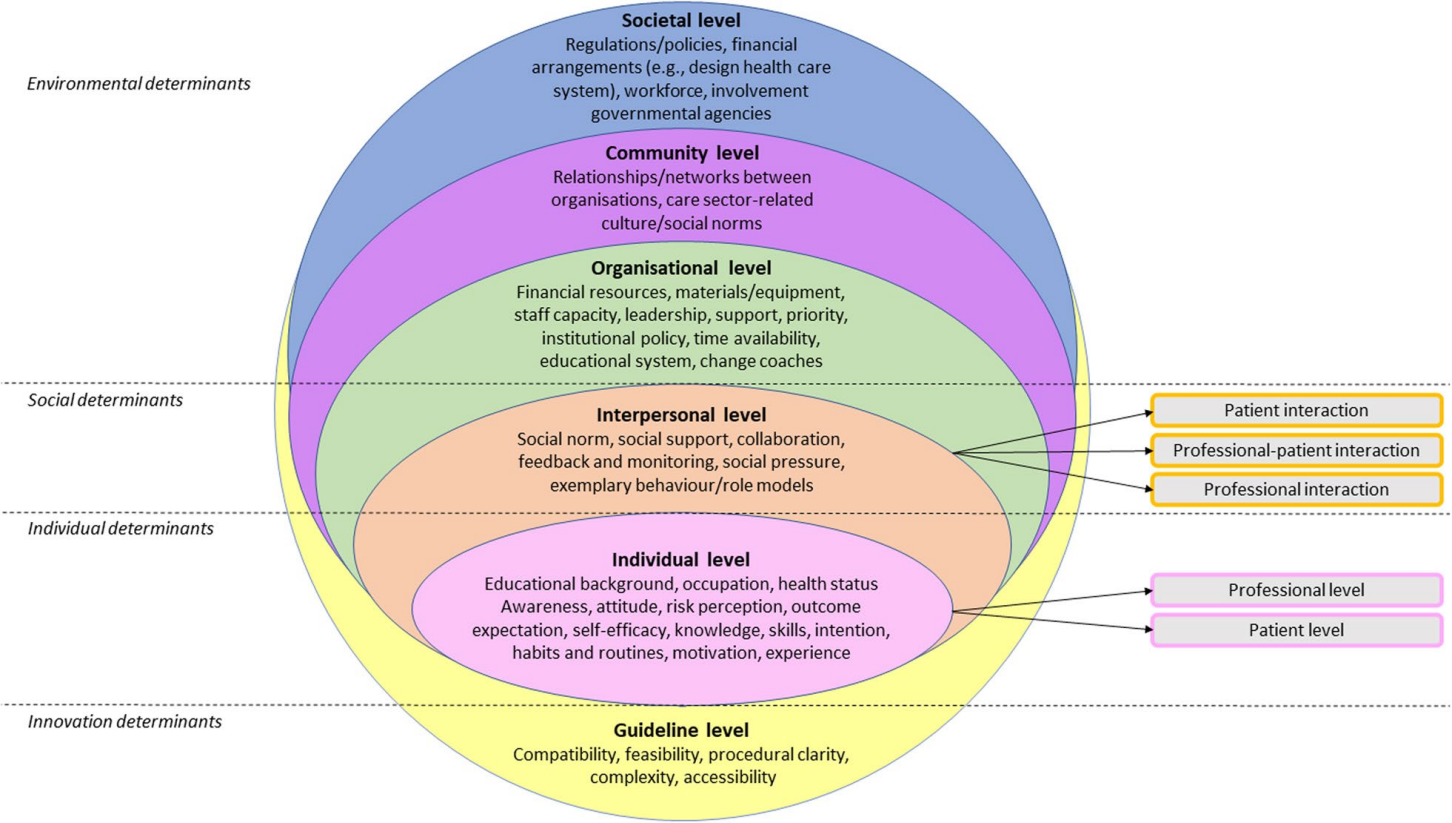
The integrated theoretical framework for factors influencing IPC in the mental health care setting, guided by our pre-proposed integrated theoretical framework for factors influencing IPC [18] and its underlying implementation science theories [23–30], adapted to the results of our qualitative analysis

Based on the results of our qualitative analysis, we developed an integrated theoretical framework for factors influencing IPC in the mental health care setting (Fig. 1) to underpin our data mapping and reporting.

*The integrated theoretical framework includes the guideline (yellow), individual (pink), interpersonal (orange), organisational (green), community (purple), and societal level (blue). The individual level comprises the patient and professional level. The interpersonal level includes professional interaction, professional-patient interaction, and patient interaction. The division of levels is based on our pre-proposed theoretical framework for factors influencing IPC *[18]*. The underlying concepts of every level are based on various implementation science theories *[23–30]*, adapted to the data from our qualitative analysis.*

### Perceived barriers and facilitators to IPC

Perceived barriers and facilitators will be discussed per level and theme on which they appeared. An overview of all barriers and facilitators is provided in Table 1, displayed per level, and categorised by corresponding theme. Quotations that included rich descriptions and/or contextual depth were used to support the findings.

**Table 1:**
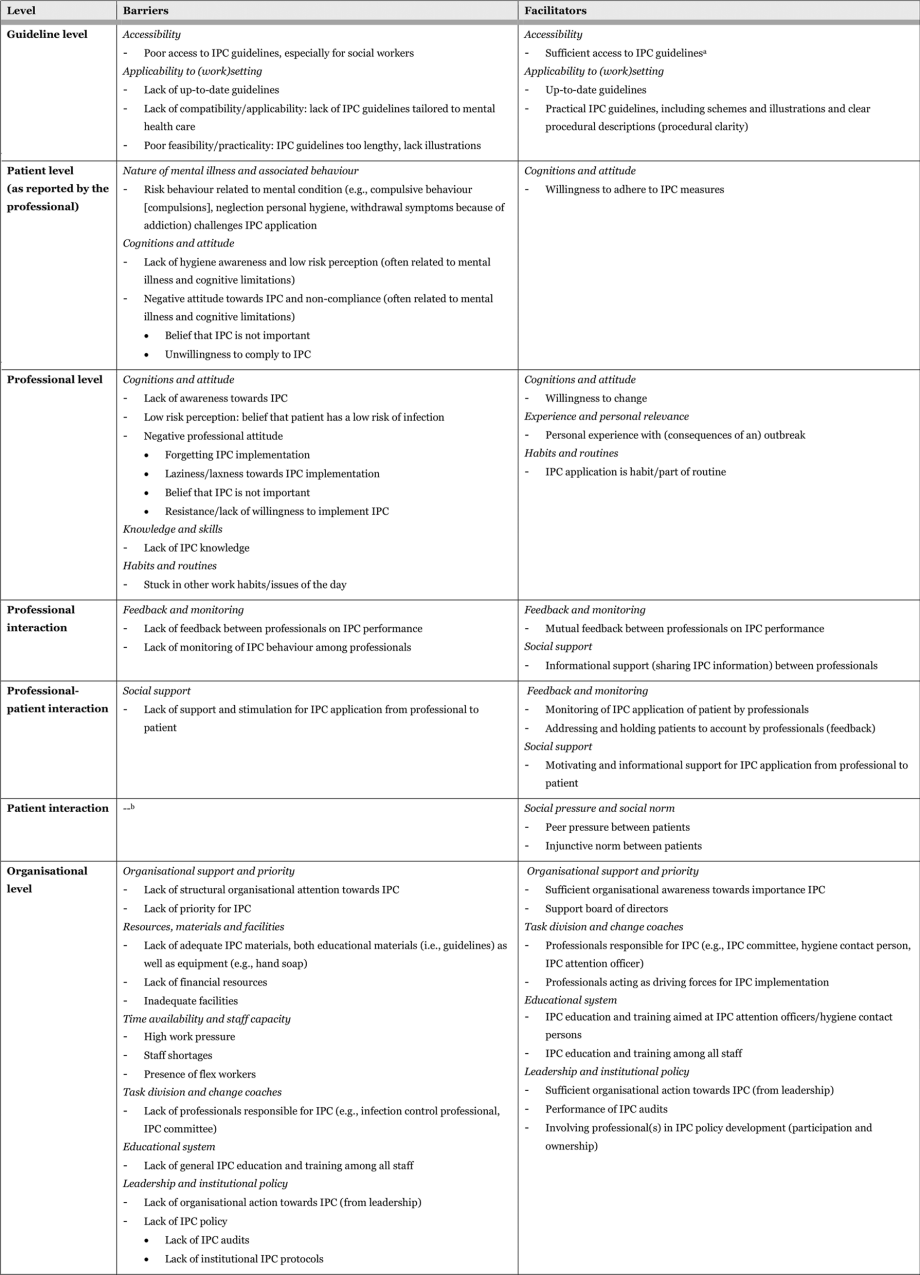

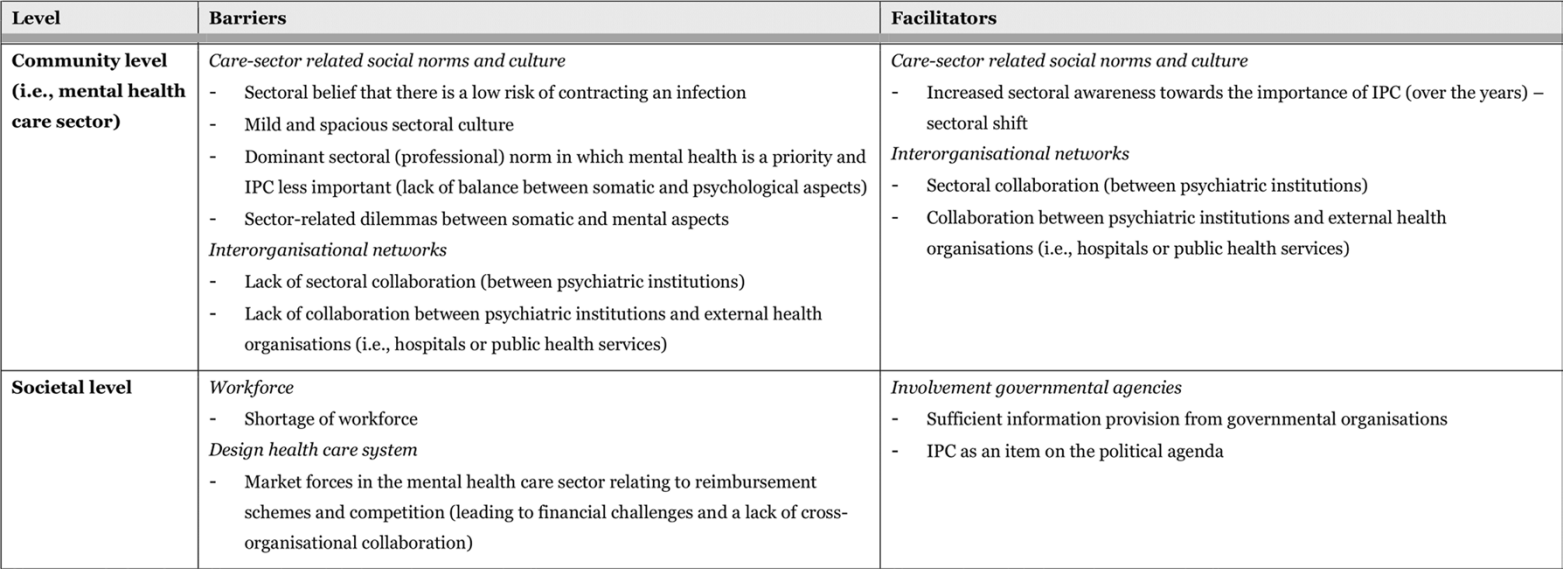
Barriers and facilitators to IPC implementation, perceived by professionals working at psychiatric institutions (n = 16), depicted per level of the integrated theoretical framework (see Fig. 1), and categorised by corresponding theme

^a^ Although guidelines are accessible, and known, its application depends on human action and professional factors

^b^ No barriers were reported for the domain ‘patient interaction’

*IPC* infection prevention and control

Note. Concepts in italics are the themes which categorised the perceived barriers and facilitators

### Guideline level

#### Accessibility

The majority of participants mentioned guidelines were easily accessible, often via an online portal. Still, social workers indicated they lack access to guidelines:* “I am not familiar with existing IPC guidelines, but I assume we have them. However, it is never discussed where to find them, so I do not know.” (P14, woman, 45–50y, social worker).* Participants recognised although IPC guidelines were accessible, they were only used in case of an outbreak and not in a preventive manner: *“IPC guidelines are in our quality manual. They are only used when necessary, in case of an outbreak. I would like them to be used more preventively.” (P5, woman, age unknown, quality assurance officer).* Some participants reported that even though guidelines were accessible and known, professionals do not apply them: *“The problem is that everyone knows we have protocols, and where to find them, but not act on them.” (P4, man, 55–60y, supervisor).* Factors on the guideline level are therefore highly dependent on professional factors.

#### Applicability to (work) setting

Frequently reported guideline-related facilitators were the presence of clear, up-to-date, and practical guidelines: *“Our current guidelines are very practical and clear because they include text, pictures, and clear steps.” (P8, woman, 25–30y, manager)*. When these characteristics were absent in guidelines, participants perceived it as a barrier. In addition, one participant mentioned current guidelines lacked practicality/feasibility since they include too much information. A few participants indicated a lack of guidelines specifically tailored to mental health care:* “Existing guidelines are quite general, applying to everyone. They should be adapted to staff members’ profession and patient groups. Because in the workplace, you often get little use out of current guidelines.” (P1, man, 65–70y, physician).*

### Patient level

#### Nature of patients’ mental illness and associated behaviour

A central patient-related issue is the nature of a patient’s mental illness and the risk behaviour associated with their condition, which often challenges IPC application: *“When individuals have been diagnosed with schizophrenia for twenty years or more, they are not concerned with themselves nor have room for it in their heads. Some people are completely contaminated due to a lack of personal hygiene.” (P3, woman, 40–45y, nurse),* “*We have one patient who constantly picks her nose and then touches everything, which has to do with the compulsions of her mental illness. This may cause spread across the entire department, then you are already behind 1–0.” (P13, woman, 35–40y, nurse).* In addition, participants stated that the complex nature of patients may also interfere with IPC measures: *“At some departments, we cannot hang or display pumps of disinfectants since some patients will drink it.” (P2, woman, 55–60y, manager).*

#### Patients’ cognitions and attitude

Other frequently reported barriers on the patient level are the prevalence of low hygiene awareness and a negative attitude towards IPC (often related to patients’ mental illness and cognitive limitations), resulting in non-compliance among patients: *“We have a lot of patients that are unwilling and non-compliant. They do not see the importance. Sometimes it is unwillingness, other times inability. They are just in their own heads with their own perceptions. Also, sometimes it is just laziness.” (P3, woman, 40–45y, nurse).* Nevertheless, some participants mentioned patients are in the end willing to apply IPC measures: “*When patients show reluctance, we often mention they are expected to shower or clean their room. In general, when we tell patients that, they pick up things reasonably well.” (P14, woman, 45–50y, social worker).*

### Professional level

#### Professionals’ cognitions and attitude

On the professional level, a lack of awareness, low risk perception and a negative attitude were often mentioned as important barriers: *“A change in attitude is needed among colleagues so they become more alert, in a way that IPC becomes integrated into their professional attitude and awareness. People often believe there is no risk.” (P1, man, 65–70y, physician).* These factors emerge from the belief that IPC is only related to technical nursing activities, which prevails among non-nurses. The interviews showed a difference in cognitions and attitudes between different professionals. Especially nurses acknowledged the importance of IPC and indicated that a lack of awareness and a negative attitude regarding IPC are often prevalent among psychiatrists and social workers. This is attributed to the educational background: *“I attach great importance to IPC and pay more attention to it than colleagues from other sectors. Hygiene and IPC was a recurring theme during my educational programme, and I think that lacks in for example social work studies, which results in less awareness and alertness.” (P10, woman, 20–25y, nurse).* One participant perceived a lack of hard evidence for IPC: “*I doubt to what extent some IPC measures and rules actually prevent the spread of infections. For example, I doubt whether wearing a white coat as a physician is more hygienic than wearing own clothes. I would like to see the findings of a comparative study.” (P15, man, 55–60y, psychiatrist)*. Nevertheless, a single participant reported that some professionals are willing to change and implement IPC, which is considered to be facilitating.

#### Professionals’ knowledge and skills

Participants often mentioned knowledge deficits as important barrier: “*Not everyone has the same level of knowledge. People who have always worked in psychiatry have less knowledge of somatic aspects; they only have eye for the psyche.” (P3, woman, 40–45y, nurse).*

#### Professionals’ habits and routines

Participants commonly perceived that other work issues and routines could hinder IPC: *“You notice that a lot of things go in the rush of the day, and people do things quickly and without thinking. That is where things go wrong, or something is forgotten. IPC often does not have the highest priority in the issues of the day.” (P8, woman, 25–30y, manager).* Nonetheless, existing routines and habits may also facilitate IPC application: *“It is nice to see that nowadays employees always wear gloves when they are in contact with vulnerable patients. Recently an employee said 5 years ago she would have found it strange to wear gloves but now she thinks it is weird not to wear gloves.” (P5, woman, age unknown, quality assurance officer).*

#### Professionals’ experience and personal relevance

Several participants indicated that experiencing an outbreak and its associated negative consequences may positively influence professionals’ perceptions and therefore IPC behaviour: *“Things are changing, we have had several outbreaks, so awareness skyrocketed. The point is, there are relatively few infections and outbreaks in our sector, so you would almost hope something would go wrong sometimes since it is the best way to make everyone face the facts.” (P6, man, age unknown, supervisor)*. In line, a few participants reported IPC generally receives little attention at the workplace, until practice makes it relevant: *“IPC is not something we pay attention to in our daily work. It only becomes relevant when we are faced with a severe infection or outbreak.” (P12, woman, 55–60y, supervisor).*

### Professional interaction

#### Feedback and monitoring between professionals

Participants often reported feedback between professionals on IPC performance as a facilitator: *“It is very important that colleagues hold each other accountable regarding their behaviour and address each other when something is not performed well.” (P13, woman, 35–40y, nurse)*. At the same time, participants acknowledged a lack of mutual professional feedback, and stated that professionals do not monitor each other’s IPC behaviour: *“I think 99 out of 100 times we would not address each other or hold each other accountable. That is not our culture.” (P7, man, 45–50y, psychiatrist), “People do not check each other’s IPC behaviour, even our nurse who has IPC knowledge does not address colleagues.” (P14, woman, 45–50y, social worker).*

#### Social support between professionals

Several participants experienced social support between professionals, in the form of informational support, as facilitating: *“When something is unclear to employees, for instance with regard to guidelines, explanation is provided by our task group and practice assistants are often the contact person who provide information. That kind of assistance and support is important.*” *(P1, man, 65–70y, physician).*

### Professional-patient interaction

#### Social support from professional to patient

An important facilitator concerning the professional-patient interaction is social support from professionals towards patients, in the form of motivating and informational support: “*I try to stimulate patients to adhere to hygiene and IPC measures. It is important and I also like to explain to patients why those things are important.” (P10, woman, 20–25y, nurse).* However, some participants reported social support from professionals towards patients is often lacking: *“Stimulating patients to apply hand hygiene and other IPC measures differs per department and employee. It is very important but often performed too little.” (P13, woman, 35–40y, nurse).*

#### Feedback and monitoring between professional and patient

Frequently discussed facilitators regarding professional-patient interaction are the monitoring of a patient’s IPC application by the professional and the provision of feedback in case of non-adherence: *“If patients do not wash their hands after a toilet visit, we send them back. As employees, we address patients on their hand hygiene. Especially since personal hygiene is not a top priority for them, we need to pay attention to it as a department.” (P8, woman, 25–30y, manager).*

### Patient interaction

#### Social pressure and social norm between patients

Regarding patient interaction, a single participant indicated peer pressure between patients could facilitate IPC compliance among patients: *“Because we work and live in a group context, peer pressure is often helpful when a patient finds something less important.” (P14, woman, 45–50y, social worker).* In line, a few participants reported that calling upon the social norm, in particular the injunctive norm, of patients may be helpful: “*A lot of times when people do not want to shower, we tell them that other people are bothered by it. Patients are sensitive to that and as a result cooperate.*” *(P4, man, 55–60y, supervisor)*.

### Organisational level

The majority of identified factors that could hamper or facilitate IPC were found at the organisational level.

#### Organisational support and priority

In general, participants reported sufficient organisational awareness towards the importance of IPC. A number of participants perceived the support of the board of directors as sufficient which positively influences IPC: *“The organisation is increasingly aware of the importance of hygiene and that we have to work hygienically. The board of directors actively supports this. I believe the board of directors and management must stand behind it, otherwise IPC will not make it.” (P5, woman, age unknown, quality assurance officer).* However, participants indicated that although support may exist, IPC lacks priority and receives no structural organisational attention: “*IPC only receives attention when it is relevant. IPC is not prioritised enough. But if there were to be an outbreak, all attention will be focused on it.” (P10, woman, 20–25y, nurse).*

#### Resources, materials and facilities

A frequently reported organisational barrier is a lack of adequate materials, facilities, and financial resources: *“It is a very old building which impedes hygiene.” (P12, woman, 55–60y, supervisor), “IPC costs money. Money is a limiting factor, especially for managers.” (P3, woman, 44y, nurse), “We do not have the right equipment everywhere, such as paper towels and soaps.” (P2, woman, 55–60y, manager).* In addition*,* a few participants reported a general lack of educational materials such as IPC guidelines.

#### Time availability and staff capacity

High work pressure, staff shortages, and the presence of flex workers are perceived to be important hindering factors: “*There is too little time to implement IPC sufficiently. There are time and staff shortages.” (P10, woman, 20–25y, nurse)*, *“Due to flex workers it is difficult to properly implement IPC, since they do not have the same attitude as permanent employees and are less likely to address patients with respect to hygiene because they do not know how patients will react.” (P5, woman, age unknown, quality assurance officer).*

#### Task division and change coaches

Participants frequently indicated the presence of a person or group responsible for IPC—such as a hygiene contact person, IPC attention officer, IPC committee—as a facilitator: *“Something that promotes IPC is when contact persons are present at every location, and employees know of their existence and make use of them. That is our gain.” (P1, man, 65–70y, physician), “IPC attention officers are selected and educated, and they disseminate their knowledge. In doing so, IPC received more attention.” (P13, woman, 35–40y, nurse).* Organisations often ensure task division among their staff members, in which a person or group is appointed to coordinate and sustain IPC, and functions as first point of contact. This is perceived as facilitating since task division results in tailor-made policy: “*It is an illusion to keep all professionals informed about IPC at all times, knowledge fades.” (P1, man, 65–70y, physician), “We try to complement each other, so the fact that not everyone knows everything is not a problem.” (P3, woman, 40–45y, nurse).* Some participants noted a lack of professionals responsible for IPC in their organisation, yet indicate the need: *“At the moment, there is no one in our organisation that deals with IPC in particular. It would be of added value if we could establish that.” (P12, woman, 55–60y, supervisor).* A frequently reported facilitator is the presence of professionals who act as driving forces for IPC implementation: *“IPC depends on whether someone is motivated, then it gets implemented, otherwise it is ignored (…) It is important to keep sounding the alarm bell; insist it has to happen. Then you slowly observe that more and more attention is paid to it and people are increasingly realising its importance.” (P5, woman, age unknown, quality assurance officer).*

#### Educational system

Participants expressed a general lack of IPC education and training among all staff within their organisations. They noted IPC education is currently mainly available to IPC attention officers/hygiene contact persons: “*Our hygiene contact person received training from the Public Health Service about what infections are, how to prevent them and how to deal with measures at the workplace.” (P1, man, 65–70y, physician).* The education of attention officers/hygiene contact persons is important since they disseminate knowledge among their co-workers. Yet, participants indicated the need for general education provision among all staff: “*It would be nice if people would come by to educate us on guidelines and common infections and how to prevent these. There is a need for education, it would be of added value.” (P12, woman, 55–60y, supervisor), “Everyone should be involved in trainings, also non-nurses” (P9, woman, 35–40y, nurse).*

#### Leadership and institutional policy

A few participants indicated sufficient organisational action from leadership: *“Every time I address something to our management, they undertake action.” (P5, woman, age unknown, quality assurance officer).* Nevertheless, participants occasionally reported a lack of organisational action: *“We often sound the alarm bell, but then no action is taken. We address certain aspects more than once, but then it gets sidetracked. They look at it, but then it disappears, no idea where.” (P16, woman, age unknown, nursing assistant).* Some participants indicated their organisation lacks policy regarding IPC, including the performance of IPC audits, whereas this is perceived as facilitating for IPC: *“Audits make sure everyone is more alert.” (P8, woman, 25–30y, manager)*. In addition, a few professionals reported a lack of IPC protocols within their organisation. A single participant reported that involving professionals in IPC policy development is facilitating: *“Our organisation wants to involve professionals, to think along. It is very good that we are asked to contribute ideas about what we consider important.” (P3, woman, 40–45y, nurse).*

### Community level

#### Care sector-related social norms and culture

Most participants acknowledged psychological aspects to be the core concern within mental health care, whereas somatic aspects (including IPC) are less important. Several participants perceived this as a barrier: “*We are psychiatry, so that is everyone’s priority, not IPC. That should go together more often because you cannot see one separately from the other.” (P5, woman, age unknown, quality assurance officer).* A subtheme therein is the overall low risk perception regarding infections in the sector: *“The issue is to convince people that within mental health care we are just as much at risk of infections as any other sector.” (P6, man, age unknown, supervisor).* In line, some participants noted the dominant ‘mild and spacious’ culture in the sector may hinder IPC:* “Mental health care is characterised by a culture in which people are little concerned with all kinds of rigid, authoritative norms and values. There is a culture of space, understanding and relaxedness, which hinders strictness such as IPC.” (P7, man, 45–50y, psychiatrist).* Participants moreover recognised there is often a dilemma between somatic and mental aspects, which challenges IPC:* “In mental health care you look on the one hand from a somatic point of view since we are caregivers and on the other hand you look from a psychological point of view. For example, isolation of an infected person is needed in the eye of risk infection, but in contrast with mental aspects.” (P10, woman, 20–25y, nurse).* Yet, several participants pointed to a shift of vision in the sector, in which IPC received more attention recent years: “*The positive thing is that increasingly more attention is paid to IPC. Awareness is increasing. Five years ago, it was not even possible to demand soap.” (P2, woman, 55–60y, manager).*

#### Interorganisational networks

Collaboration between psychiatric institutions as well as collaboration with external health organisations—such as hospitals or public health services—is perceived as facilitating: “*Public health services have a lot of guidelines, so we would like to work together. But we would also like to know from fellow psychiatric institutions, how they approach IPC and what challenges they encounter.” (P4, man, 55–60y, supervisor)*. Despite the need for cross-organisational collaboration, some participants indicated a general lack thereof, which is related to market forces in the sector:* “When we need advice, we often call a medical microbiologist from the hospital. But the moment practical questions come up, they direct us to an infection control professional. That used to be very easy but nowadays they say: but we do not have a contract, do we?” (P2, woman, 55–60y, manager), “As psychiatric institutions we have little contact with each other, which is due to mutual competition in the sector.” (P12, woman, 55–60y, supervisor).*

### Societal level

#### Design health care system

A regularly reported issue is the way health care costs regarding mental health services are currently reimbursed, controlled and constrained in the Netherlands. Participants indicated current reimbursement schemes hinder IPC implementation due to financial challenges: *“At the moment, the entire sector is having a hard time. It is very difficult to get reimbursed for treatments we provide. Therefore, we lack financial resources, leaving little room for extra things such as IPC. The psychological part is the most important since it provides us money.” (P12, woman, 55–60y, supervisor).* The current system of mental health service could also indirectly hamper IPC implementation. Participants argued that market forces in mental health care impede cross-organisational collaboration.

#### Workforce

A second barrier related to societal influences is the workforce shortage: *“There are no nurses available, so when they apply for a job and have a great resume but red nails, a facility is not going to complain about nails.” (P2, woman, 55–60y, manager).*

#### Involvement governmental agencies

An occasionally reported facilitator is sufficient information provision concerning IPC from governmental organisations. A single participant moreover reported IPC in mental health care becoming an item on the political agenda as facilitating: *“It also helps when IPC is on the political agenda. For somatic hospitals and nursing homes this has been going on for some time now. I am glad attention is also brought to IPC in mental health care.” (P2, woman, 55–60y, manager).*

### Recommendations reported by professionals to improve IPC

Adding to previously suggested recommendations by professionals (as described above) to promote IPC, such as to increase awareness towards IPC among all staff members, participants reported specific methods in which this can be achieved. As previously indicated, participants frequently recommended introducing education and training for all personnel, or general information provision: “*More awareness is needed, which can be enhanced by offering courses, or by providing information during work meetings or email.” (P6, man, age unknown, supervisor),* “*By providing information you hope awareness towards the importance of IPC will permeate into the attitudes of professionals of all disciplines. Not only among physicians or nurses but also among psychologists, social workers and cleaning staff.” (P1, man, 65–70y, physician).* In addition, to increase awareness, participants regularly advised the communication of formal agreements within the institution: *“Protocols should be discussed and communicated, or at least be on the agenda every now and then. Now it is never talked about.” (P14, woman, 45–50y, social worker).* Some participants recommended to establish institutional IPC protocols in case these were absent: “*Protocols have to be drawn up, thereby, we can increase awareness among all employees.” (P11, man, 50–55y, nurse).* Nevertheless, these institutional protocols should be mindful of the specific contextual factors characterising mental health care.

Another frequently reported recommendation is to make room for and facilitate IPC on the organisational level, by reducing work demands and providing adequate equipment: *“Staff should feel they have enough room in their heads and agendas to implement IPC. IPC should not be an extra task on top of all other tasks. While our productivity, the number of consultations you have per day, has to be 80%.” (P7, man, 45–50y, psychiatrist), “Something that needs to change is the workload and high work pressure. They have to facilitate IPC and make it as easy as possible, by ensuring all necessary equipment is available.” (P13, woman, 35–40y, nurse).* As noted earlier, professionals recommended to establish task division by appointing a professional responsible for IPC: *“It would help if someone became responsible for IPC. Someone who specifically deals with this. A point of contact who can also take measures and decisions and monitors whether these are met.” (P4, man, 55–60y, supervisor).* Participants suggested this professional could also assist in increasing awareness and keeping professionals up-to-date with new insights regarding IPC practices.

A central theme emerging among participants is the need for a comprehensive approach to improve IPC. Participants acknowledged it is not sufficient to implement one component or facet, but efforts should be complemented: *“We should adopt a comprehensive approach, you have to facilitate and sustain IPC, but you also have to educate new employees and it should also become a theme in the education and training of physicians, psychiatrists and other mental health care professionals.” (P2, woman, 55–60y, manager).* Another central matter deriving from the interviews is the need for a tailored approach to implement IPC in mental health care: *“We must develop tailor-made policy; looking at what are we going to offer, facilitate or even make mandatory in which situation? We have to implement hand hygiene and work hygienically, but to what extent should we impose IPC in a department where we have to accommodate a domestic atmosphere?” (P2, woman, 55–60y, manager).* Another occasionally reported recommendation is to implement audit and feedback: *“It would be of added value if someone would visit every department to assess and reflect on what grade everyone gets for IPC and which important improvements to make.” (P10, woman, 20–25y, nurse).* In addition, the inclusion of hygiene in patients’ treatment protocol is at times recommended:* “We should include hygiene in the treatment protocol, then it will receive more attention.” (P4, man, 55–60y, supervisor).* Furthermore, a few participants recommended including IPC as a structural educational element in the curriculum of study programmes (in both medical and social work-oriented studies).

## Discussion

This study assessed perceived barriers and facilitators to IPC in psychiatric institutions, which was informed by various implementation science theories [23–30] and guided by our pre-proposed integrated theoretical framework [18]. Our qualitative analysis identified six main themes: patients’ non-compliance (strongly related to mental illness); professionals’ negative cognitions and attitude towards IPC and IPC knowledge deficits; monitoring of IPC performance between professionals and mutual professional feedback; social support from professional to patient; organisational support and priority; and financial and material resource limitations (related to financial arrangements regarding mental health services).

Professionals also provided recommendations to improve IPC. The main recommendations included: (1) to increase awareness towards IPC among all staff members, by education and training, and the communication of formal agreements as institutional IPC protocols; (2) to make room for and facilitate IPC at the organisational level, by providing adequate IPC equipment and appointing a professional responsible for IPC.

We identified apparent challenges between IPC, mental health treatment needs of patients and the overall insubstantial role of IPC in mental health care settings. This dilemma between providing good mental health care and ensuring adequate IPC practice may affect professionals’ cognitions and attitudes, and the degree of organisational priority. This parallels a review that indicated psychiatrists often consider the treatment of mental illness as their primary task, thereby often disregarding risks of physical illness [39]. Another qualitative study highlighted a lack of understanding of effective IPC procedures and the undervaluation of IPC importance among mental health professionals [40]. These findings moreover align with other studies concluding that mental health staff is less familiar with effectively managing infectious diseases compared to other HCWs [15, 17]. Our findings revealed social support from professional to patient as an important theme. This is in line with qualitative findings that indicate the implementation of effective IPC practices in psychiatric settings rely on collaboration between professionals and patients [40]. Other main findings of our study are financial and material resource limitations, which is in accordance with an observational study that recognised psychiatric facilities often have few (financial) resources, personnel, and diagnostic measures to implement IPC, leading to low adherence to IPC practices [5]. Moreover, several studies have stated that patients with mental illness often do not care for themselves regarding hygiene [3, 5, 7] and are often non-compliant [40], which parallels our findings on the patient level.

Previous research has indicated that the development and implementation of evidence-based guidelines is a prerequisite for effective IPC practice [39]. Nevertheless, a Cochrane review reported that only modest improvements can be expected in practice when using only printed educational materials such as guidelines [41]. Successful IPC requires next to the presence of guidelines, coordinated efforts at national and institutional levels, including clear communication [42].

Previous studies conducted in hospital and long-term care settings have suggested that multifaceted interventions—including system change, training and education, monitoring and feedback, reminder and communication, and enabling work culture—are effective in promoting and sustaining IPC compliance [43–45]. We also highlight the importance of adopting a comprehensive and multilevel approach to optimise IPC in psychiatric settings. To effectively implement IPC in mental health care settings, psychiatric facilities should adopt strategies that ensure a good balance between psychological and physical aspects of care [46].

### Strengths and limitations

A major strength of this study is the theory-informed approach. The theoretical framework guiding this study recognises the existence of factors on multiple levels, therefore enabling a rigorous discussion and providing a viewpoint which moves beyond the use of mere simplified behaviour change theories. Furthermore, previous studies have indicated that social, cultural, and organisational factors influencing implementation behaviour are rarely considered when translating strategies into practice [47]. This highlights the relevance of our findings since they account for these factors and can inform IPC practice improvements. A second strength is represented by the coding process which was independently conducted by two trained researchers. Qualitative analysis was moreover reviewed by a third researcher, which enhanced the quality of data analysis.

A potential limitation of this study is the use of snowball sampling. This convenience sampling method may have resulted in some selection bias, since participants might have been the most enthusiastic individuals within the organisations. Nevertheless, our sample included professionals from various types of occupations and from various layers of the organisation, hereby presumable being representative of the study population. Another limitation is the performance of data analyses after conducting all interviews. Consequently, insights from data analyses did not direct the content of subsequent interviews. Another limitation is that barriers and facilitators at the patient level were identified by professionals, and the patient perspective was not obtained.

One should remark that the mental health care system may differ between countries, indicating that the findings regarding financial limitations might need careful consideration. Nevertheless, mental health service provision, and the related financial arrangements and reimbursement schemes, are similar to some extent throughout Western world countries.

## Implications for practice and conclusion

The sustained implementation of IPC is challenging in psychiatric settings, yet crucial to protect the vulnerable patient population. The COVID-19 pandemic magnified the recognition of the importance of IPC in care facilities, which assumably increases support and commitment to promote and improve IPC implementation. Thereby, increasing the relevance of our results for care practice.

Based on our main findings, an initial recommendation for practice is to appoint a professional who is responsible for IPC. For this, we recommend a two-level structure: (a) an infection control professional and (b) an IPC attention officer. An infection control professional is a trained expert in IPC and leads the planning, development, implementation, coordination, and evaluation of IPC policy in (health)care facilities, including the provision of IPC education to staff members and the performance of IPC audits. Infection control professionals work ‘behind the scenes’ rather than in direct patient care. On a more team or department level, IPC attention officers promote IPC awareness among fellow staff members, serve as a (first) point of contact and function as a link between the workplace and infection control professional.

In this study we showed that an integrated theoretical framework for factors influencing IPC [18] can guide the examination of factors influencing IPC in various institutional care contexts including psychiatric institutions. We present an integrated theoretical framework for mental health care settings. Application of this framework may inform researchers and policymakers to develop theory-informed interventions.

To conclude, the implementation of IPC in psychiatric institutions is strongly influenced by factors on the patient (e.g., noncompliance), professional (e.g., attitudes and knowledge) and organisational (e.g., priority, financial resources, and IPC equipment) level. Professional interaction (e.g., mutual professional feedback) and professional-patient interaction (e.g., social support from professional to patient) appeared to be additional important aspects. Coordinated actions at the institutional level using multidimensional approaches will be required for success. Moreover, future IPC practice improvements should ensure a balance between mental health care and IPC needs to sustain IPC.

## Supplementary Information

**Additional file 1. **COREQ (COnsolidated criteria for REporting Qualitative research) Checklist.

**Additional file 2. **Interview topic guide.

**Additional file 3. **Example of the coding process.

## Data Availability

The datasets used and/or analysed during the current study are available from the head of the data-archiving of the Public Health Service South Limburg on reasonable request. Interested researchers should contact the head of the data-archiving of the Public Health Service South Limburg (Helen Sijstermans: helen.sijstermans@ggdzl.nl) when they would like to re-use data.

## Abbreviations

HAIs: Healthcare-associated infections
COVID-19: Coronavirus disease 2019
HIV: Human immunodeficiency virus
AMR: Antimicrobial resistance
HCWs: Healthcare workers
IPC: Infection prevention and control
COREQ: COnsolidated criteria for REporting Qualitative research
