# Correction to: Barriers and facilitators to infection prevention and control in Dutch psychiatric institutions: a theory-informed qualitative study

**DOI:** 10.1186/s12879-022-07318-1

**Published:** 2022-04-21

**Authors:** Famke Houben, Mitch van Hensbergen, Casper D. J. den Heijer, Nicole H. T. M. Dukers-Muijrers, Christian J. P. A. Hoebe

**Affiliations:** 1 Department of Sexual Health, Infectious Diseases and Environmental Health, South Limburg Public Health Service, P.O. Box 33, 6400 AA Heerlen, The Netherlands; 2 Department of Social Medicine, Care and Public Health Research Institute (CAPHRI), Faculty of Health, Medicine and Life Sciences, Maastricht University, P.O. Box 616, 6200 MD Maastricht, The Netherlands; 3 Department of Medical Microbiology, Care and Public Health Research Institute (CAPHRI), Faculty of Health, Medicine and Life Sciences, Maastricht University Medical Centre (MUMC+), P.O. Box 5800, 6202 AZ Maastricht, The Netherlands; 4 Department of Health Promotion, Care and Public Health Research Institute (CAPHRI), Faculty of Health, Medicine and Life Sciences, Maastricht University, P.O. Box 616, 6200 MD Maastricht, The Netherlands

## Correction to: BMC Infectious Diseases (2022) 22:243 10.1186/s12879-022-07236-2

Following publication of the original article [1], the authors identified some errors in Table 1. The correct Table 1 is given below:

**Table 1:**
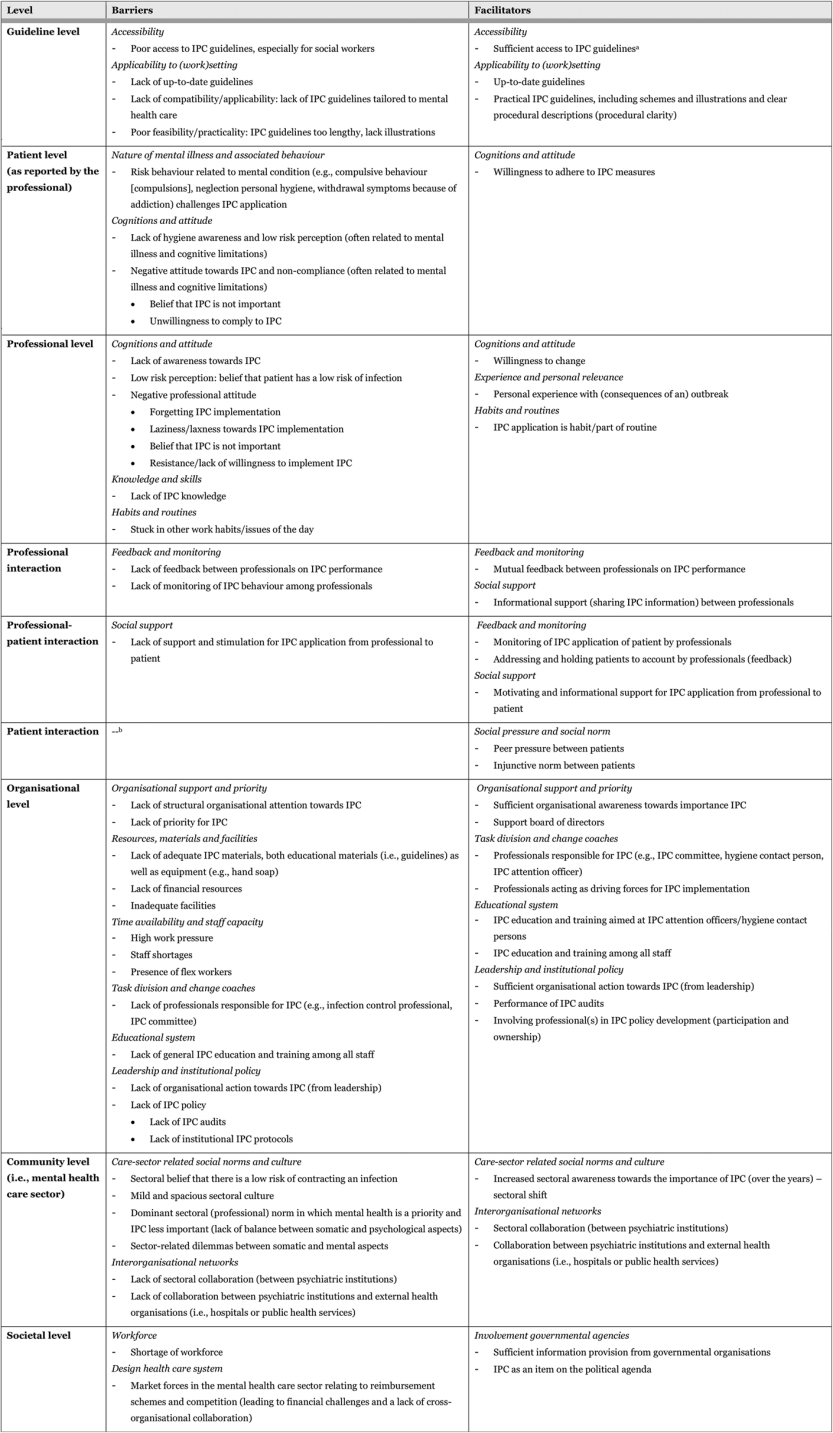
Barriers and facilitators to IPC implementation, perceived by professionals working at psychiatric institutions (n = 16), depicted per level of the integrated theoretical framework (see Fig. 1), and categorised by corresponding theme

Furthermore, the authors noticed a typo in the section ‘implications for practice and conclusion’: “theo ry-informed interventions” should be replaced with ‘theory-informed interventions’.

The original article [1] has been corrected.
