# Supplementary material for: Barriers and facilitators to infection prevention and control in Dutch psychiatric institutions: a theory-informed qualitative study
Source: BMC Infect Dis. 2022 Mar 11;22:243. doi: 10.1186/s12879-022-07236-2 (PMC8914451; doi:10.1186/s12879-022-07236-2)
Supplement: Supplementary file 2 — Additional file 2. Interview topic guide. [file 12879_2022_7236_MOESM2_ESM.pdf]

# Additional file 2: Interview topic guide

| | |
|---|---|
| **Background variables** | 1. Could you introduce yourself?<br>o Age, gender, occupation, (work) experience. |
| | 2. Could you tell something about your work? What are your daily work-related responsibilities? |
| **Influencing factors (i.e., barriers and facilitators)** | 3. What comes to mind when you think about infection prevention and control (IPC)?<br>o To what extent do you consider IPC important?<br>o What role did IPC play during your education/studies?<br>o Does your institution offer IPC education/are you taking IPC courses or training?<br>o Are you aware/familiar with IPC guidelines (e.g., hygiene guidelines) in your institution?<br>i. To what extent do these guidelines play a role in your work? (hand hygiene, personal protective equipment, clothing regulations, hygienic working environment, resources and materials)<br>ii. Are these guidelines adequate? (e.g., comprehensibility, practicality)<br>o How do you acquire IPC information? |
| | 4. To what extent does IPC play a role at work?<br>o How do you think your colleagues think of (the importance of) IPC?<br>o To what extent do people check/correct each other on IPC/hygiene?<br>o Do you make a distinction regarding IPC between different patient groups (based on care needs?)<br>o To what extent do you stimulate/motivate patients to apply hygiene/IPC measures?<br>o Could you share some experiences with IPC (measures)? |

| | |
|---|---|
| | 5. To what extent does IPC play a role on an organisational level? (e.g., policy, formal agreements).<br>o How much attention is paid to IPC?<br>o Is enough priority given to IPC?<br>o Who deals with/are involved in IPC/hygiene in your organisation? |
| **Recommendations** | 6. Do you think there are changes needed with regards to IPC in your organisation? And if so, what would you recommend? |
| | 7. What needs to change so you can focus more on IPC? |
| *Abbreviation: IPC infection prevention and control* | |
