# Supplementary material for: Barriers and facilitators to infection prevention and control in Dutch psychiatric institutions: a theory-informed qualitative study
Source: BMC Infect Dis. 2022 Mar 11;22:243. doi: 10.1186/s12879-022-07236-2 (PMC8914451; doi:10.1186/s12879-022-07236-2)
Supplement: Supplementary file 3 — Additional file 3. Example of the coding process. [file 12879_2022_7236_MOESM3_ESM.pdf]

Additional file 3: Example of the coding process

**Participant**	**Quote**	**Code**	**Theme**	**Level**
Participant #7 *(man, 45-50y, psychiatrist).*	"Regarding infection prevention and hygiene, a lot of times people belief it is a waste of their time. There is not enough support for these subjects."	Negative professional attitude	Professional's cognitions and attitude	Professional
